# Serum lnc34a is a potential prediction biomarker for bone metastasis in hepatocellular carcinoma patients

**DOI:** 10.1186/s12885-021-07808-6

**Published:** 2021-02-15

**Authors:** Li Zhang, Hao Niu, Ping Yang, Jie Ma, Bao-Ying Yuan, Zhao-Chong Zeng, Zuo-Lin Xiang

**Affiliations:** 1 Department of Radiation Oncology, Zhongshan Hospital, Fudan University, 180 Feng Lin Road, Shanghai, 200032 China; 2 Department of Radiation Oncology, Shanghai East Hospital, Tongji University School of Medicine, 150 Jimo Road, Shanghai, 200120 China; 3 Shanghai East Hospital Ji’an Hospital, 80 Ji’an South Road, Ji’an City, 343000 Jiangxi Province China

**Keywords:** Hepatocellular carcinoma, Bone metastasis, Serum, lnc34a

## Abstract

**Background:**

Early screening and intervention therapies are crucial to improve the prognosis of hepatocellular carcinoma (HCC) patients with bone metastasis. We aimed to identify serum lncRNA as a prediction biomarker in HCC bone metastasis.

**Methods:**

The expression levels of lnc34a in serum samples from 157 HCC patients were detected by quantitative real-time polymerase chain reaction (PCR). Univariate analysis and multivariate analysis were performed to determine statistically significant variables.

**Results:**

Expression levels of lnc34a in serum from HCC patients with bone metastasis were significantly higher than those without bone metastasis. The high expressions of lnc34a, vascular invasion and Barcelona Clinic Liver Cancer (BCLC) stage were associated with bone metastasis by analysis. Moreover, lnc34a expression was specifically associated with bone metastasis rather than lung or lymph node metastasis in HCC.

**Conclusions:**

High serum lnc34a expression was a independent risk factor for developing bone metastasis in HCC.

**Supplementary Information:**

The online version contains supplementary material available at 10.1186/s12885-021-07808-6.

## Background

The morbidity and mortality of liver cancer continues to rise among both men and women [1]. Hepatocellular cancer (HCC), as the predominant pathological type of primary liver cancer, is the sixth most common cancer and the third leading cause of cancer deaths worldwide [2]. With the advancement of diagnostic technologies and therapeutic procedures for HCC, the overall survival has been improved in HCC patients recently [3]. However, recurrence and metastasis remain the major obstacle to reduce the mortality of HCC patients [3, 4]. The bone is the third most frequent site of distant metastasis derived from HCC, after the lungs and lymph nodes [5]. Bone colonization severely affects the life quality for HCC patients, with a median survival of 7.4 months [6]. Thus, there is an urgent need to identify potential and novel biomarkers for the early screening, diagnosing and prognosis of HCC patients with bone metastasis.

Long noncoding RNAs (lncRNAs) are a highly heterogeneous group of transcripts longer than 200 nt with limited or no protein coding capacity [7]. Many studies have shown that aberrantly expressed lncRNAs may serve as the potential biomarkers for early diagnosis, treatment, and prognosis in various cancers [8]. LncRNAs in tissue have great potential as biomarkers in cancer, while its clinical application was limited for the invasive procedure which is not easily available and may lead to complications [9].

Circulating RNA in blood is an emerging field concerned with its noninvasive diagnosis identity [10]. Up to date, more and more lncRNAs in serum or plasma have been identified as novel biomarkers for diagnosis, target therapy, and prognosis in cancers. For instance, Iempridee, T et al. (2018). identified lncRNAs AC017078.1 and XLOC_011152 as potential serum biomarkers with good diagnostic potential for cervical cancer [11]. Long noncoding RNA PCAT1, present in oesophageal squamous cell carcinoma(ESCC) cell-derived exosomes, was higher in the serum of ESCC patients and may serve as a non-invasive biomarker for ESCC [12]. LINC00899 in serum was used as a potential biomarker for the diagnosis and prognosis in acute myeloid leukemia [13]. Besides, increasing lncRNAs were identified as serum biomarkers in HCC [14–17]. For instance, the serum lncRNA uc007biz.1 (LRB1) expression levels may be considered as a novel biomarker for diagnosis and prognosis prediction of HCC, additionally complementing the accuracy of alpha-fetoprotein (AFP) and des-gamma-carboxy prothrombin (DCP) [18].

Lnc34a is a novel lncRNA with a full-length of 693 bp transcript and has no protein coding potential. It tends to be elevated in late-stage colorectal cancer. Asymmetric distribution of lnc34a during colon cancer stem cells(CCSC) division leads to the different fate of the daughter cells. Lnc34a regulated self-renewal and cell differentiation mediated by miR-34a [19], which inhibits bone metastasis in various cancers [20, 21]. Our previous study demonstrated that intratumoral Lnc34a were significantly correlated with HCC bone metastasis [22]. However, the application of circulating lnc34a in HCC patients with bone metastasis remains to be elucidated.

In the present study, we investigated the expression levels of lnc34a in serum of HCC patients using quantitative real-time polymerase chain reaction (qRT-PCR) and found that lnc34a level was correlated with HCC bone metastasis. Therefore, these findings suggested that lnc34a in serum may serve as considerable diagnostic and prognostic biomarker for bone metastasis from HCC.

## Methods

### Ethics approval and consent to participate

The study was approved by the Ethics Committee of Zhongshan Hospital, Fudan University. Informed consent was obtained from all patients prior to participation in the study according to the committee regulations.

### Patients and serum samples

All serum samples were collected from 157 HCC patients with eligibility criteria from December 2008 to October 2014. All of the 157 patients underwent curative hepatectomy in Zhongshan Hospital, Fudan University. Postoperative pathology verified HCC diagnosis. None of the patients had distant metastases at the time of blood collection. The serum was seperated from venous blood collected within 2 h and sequentially centrifuged at 4000 rpm for 10 min at 4 °C, then 12,000 rpm for 15 min at 4 °C to remove cell debris. The serum supernatants were then transferred into RNase−/DNase-free tubes and stored at − 80 °C.

The tumor stage was staged and graded according to the Barcelona Clinic Liver Cancer (BCLC) staging system and the Edmondson grading system. Liver function was assessed by the Child-Pugh scoring system. Tumor size was determined by the maximum diameter of the tumor specimen. The extent of vascular invasion was examined under the microscope in the resected specimen.

### Follow up

All patients were followed up until December 2016. The follow-up duration ranged from 3 to 96 months (mean 47 months).

All patients were followed up every 3 months after hepatectomy. The follow-up parameters were surveyed as previously described [23], including physical examinations, history documentation, ultrasonographic examination of the abdomen, a chest radiograph, laboratory tests, and so on. Bone scan or magnetic resonance imaging (MRI) was performed immediately for the patients with ostalgia. Bone metastasis was mainly diagnosed by a history of HCC, the clinical symptoms, and the imaging tests. The date from surgery to bone metastasis or death was considered as bone metastasis-free survival [23]. When the bone metastasis detected definitely, the affected bone would undergo radiotherapy. Interventional therapy, such as radiotherapy or surgery was considered for other distant metastasis.

### Ribose nucleic acid (RNA) extraction and reverse transcription quantitative polymerase chain reaction

Total RNA was extracted from serum samples using the TRIzol™ LS Reagent (Ambion, Life Technology, Carlsbad, CA, USA) according to the manufacturer’s protocol. Briefly, 0.75 ml of TRIzol LS Reagent was added for each 0.25 ml of serum sample and then 7.2*10^7^ Copies of synthesized exogenous reference λ polyA^+^ RNA (Takara, China) was added to the homogenized samples for normalization [24]. 0.2 ml of chloroform was added and the homogenized samples was centrifuged. After centrifugation, the aqueous phase was transferred to a clean tube. Then the RNA was precipitated from the aqueous phase by adding 0.5 ml of isopropyl alcohol and centrifuged. Then 1 ml of 75% ethanol was added into the tubes to wash the RNA pellet after removing the supernatant. Finally, after centrifugation, the RNA pellet was dried and dissolved with 20 μl RNase-free water. The concentration of RNA was detected by using ultraviolet spectrophotometer.

Serum RNA was reversely transcribed into Complementary deoxyribonucleic acid (cDNA) using the PrimeScript™ RT reagent kit with gDNA Eraser (Takara, China). Then qRT-PCR was performed in triplicate using SYBR® Green PCR method (Takara, China) with QuantStudio 5 Real-Time PCR System (Applied Biosystems, USA). The sequences of sense and antisense primers of lnc34a were as follows: 5′-GGAGGCTACACAATTGAACAGG-3′ and 5′-AGTCCGTGCGAAAGTTTGC-3′. The relative expression of lnc34a from serum samples was calculated using the 2^−ΔΔCT^ method and normalized against a synthesized exogenous reference λ polyA^+^ RNA.

### Statistical analyses

Statistical analyses were conducted with SPSS 24.0. Correlations between lnc34a expression and different subgroups stratified by age, gender, hepatitis B surface antigen (HBsAg), hepatitis C virus antibody (HCV-Ab), alpha-fetoprotein (AFP), alanine aminotransferase (ALT), γ-glutamyl transpeptidase (γ-GT), liver cirrhosis, Child-Pugh score, tumor differentiation, tumor size, tumor number, tumor encapsulation, vascular invasion, BCLC stage, lung metastasis, lymph node metastasis, bone metastasis were analysed by using Pearson χ^2^ test or Fisher exact test. Student’s t test was used for the quantitative analysis. Univariate and multivariate analysis were performed to determine statistically significant variables. All statistical tests were 2-tailed and a *p* < 0.05 was considered statistically significant.

## Results

### Clinicopathologic characteristics

Clinicopathologic characteristics of all HCC patients are listed in Table 1.

**Table 1:** Clinicopathological characteristics of the study population

Variable	n of patients (%)
Age	
≤ 56	78(49.7%)
> 56	79(50.3%)
Gender	
male	142(90.4%)
female	15(9.6%)
HBsAg	
negative	28(17.8%)
positive	129(82.2%)
HCV-Ab	
negative	152(96.8%)
positive	5(3.2%)
AFP	
≤ 20	60(38.2%)
> 20	97(61.8%)
ALT	
≤ 40	126(80.3%)
> 40	31(19.7%)
γ-GT	
≤ 50	96(61.1%)
> 50	61(38.9%)
Liver cirrhosis	
no	34(21.7%)
yes	123(78.3%)
Child-Pugh score	
A	151(96.2%)
B	6(3.8%)
Tumor differentiation	
I–II	93(59.2%)
III–IV	64(40.8%)
Tumor size, cm	
≤ 5	107(68.2%)
> 5	50(31.8%)
Tumor number	
single	98(62.4%)
multiple	59(37.6%)
Tumor encapsulation	
complete	72(45.9%)
none	85(54.1%)
Vascular invasion	
no	112(71.3%)
yes	45(28.7%)
BCLC stage	
0-A	99(63.1%)
B-C	58(36.9%)
Lung metastasis	
no	134(85.4%)
yes	23(14.6%)
Lymph node metastasis	
no	137(87.3%)
yes	20(12.7%)
Bone metastasis	
no	139(88.5%)
yes	18(11.5%)
Lnc34a	
low	77(49.0%)
high	80(51.0%)

*HBsAg* hepatitis B surface antigen, *HCV-Ab* hepatitis C virus antibody, *AFP* a-fetoprotein, *ALT* alanine aminotransferase, *γ-GT* γ-glutamyl transferase, *BCLC-stage* Barcelona Clinic Liver Cancer-stage

### Expression of serum lnc34a levels in patients with HCC

**Fig. 1:**
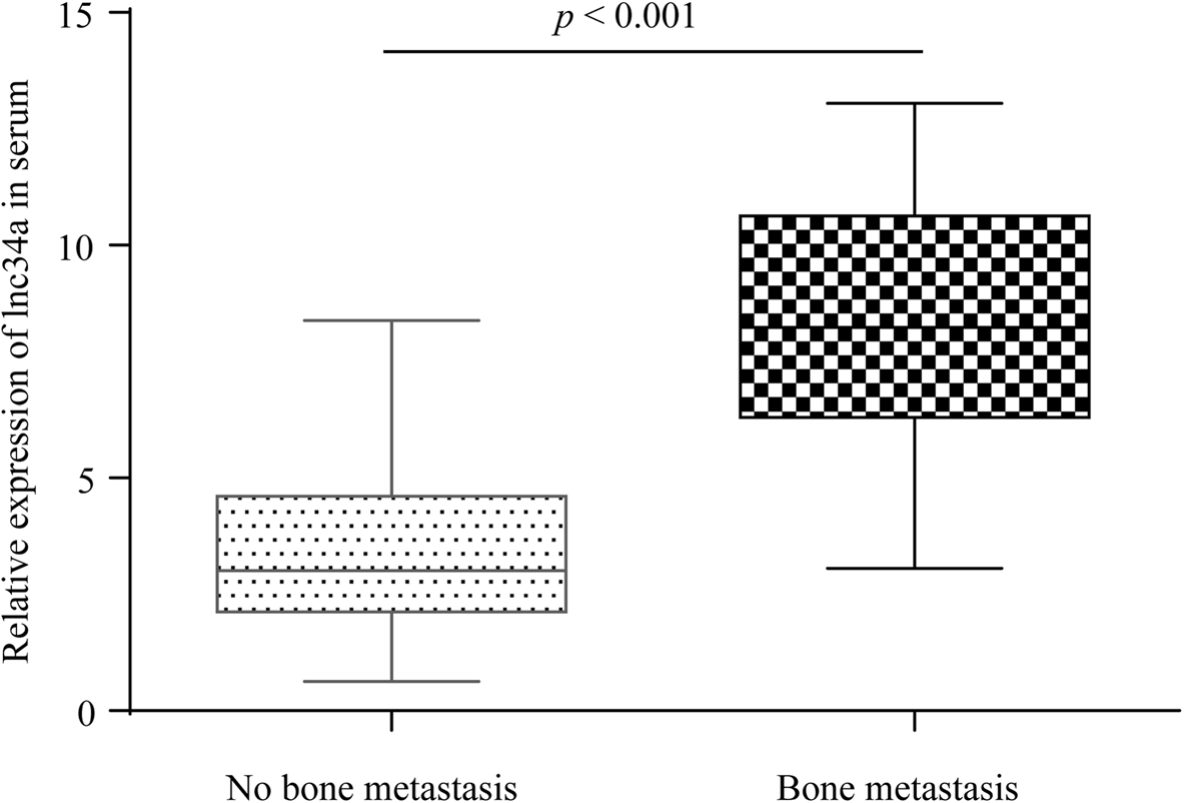
The serum levels of lnc34a in HCC patients with or without bone metastasis. Expression levels of lnc34a in serum from HCC patients with bone metastasis were significantly higher than those without bone metastasis

In order to investigate the hypothesis that the serum level of lnc34a is a potential biomarker for the bone metastases of HCC, qRT-PCR for the lnc34a expression detection was performed with serum samples from 18 HCC patients with bone metastases and 139 cases without bone metastases. As shown in Fig. 1, the serum expression levels of lnc34a in patients with bone metastases were significantly higher than that in those without bone involvement. The 157 HCC patients were classified into high serum lnc34a group (*n* = 80) and low serum lnc34a group (*n* = 77) according to the median serum lnc34a expression level [25]. Of the 18 patients with bone metastasis, 15 (83.3%) had high lnc34a expression.

Furthermore, the potential prediction value of lnc34a was evaluated by ROC curves. As shown in Additional file 1: Figure S1, the AUC of lnc34a expression was 0.683 (95% CI =0.564–0.802). The sensitivity and specificity of lnc34a to predict bone metastasis in HCC were 83.3 and 53.2%, respectively. Therefore, lnc34a might act as a useful biomarker for discriminating HCC patients with bone metastasis from those without bone metastasis.

### Association between serum lnc34a expression levels and clinicopathological factors in patients with HCC

We next analyzed the relationship between the expression of serum lnc34a and clinicopathological characteristics of the 157 HCC patients. As shown in Table 2, the analysis revealed that serum lnc34a levels were significantly related to certain clinicopathological parameters, including vascular invasion (*p* = 0.032) and BCLC stage (*p* = 0.014). Almost 82.3% patients in our study were infected with hepatitis B. However, the lnc34a expression was uncorrelated with HBsAg (*p* = 0.173), which may due to the limited number of patients in this study.

**Table 2:** Relationship between serum lnc34a expression and clinicopathological characteristics

		Serum Lnc34a Expression		
Variable	n	Low	High	P
Age				
≤ 56	78	40	38	0.577
> 56	79	37	42	
Gender				
male	142	69	73	0.727
female	15	8	7	
HBsAg				
negative	28	17	11	0.173
positive	129	60	69	
HCV-Ab				
negative	152	76	76	0.387
positive	5	1	4	
AFP				
≤ 20	60	31	29	0.605
> 20	97	46	51	
ALT				
≤ 40	126	61	65	0.749
> 40	31	16	15	
γ-GT				
≤ 50	96	49	47	0.530
> 50	61	28	33	
Liver cirrhosis				
no	34	18	16	0.608
yes	123	59	64	
Child-Pugh score				
A	151	73	78	0.643
B	6	4	2	
Tumor differentiation				
I–II	93	43	50	0.396
III–IV	64	34	30	
Tumor size, cm				
≤ 5	107	49	58	0.233
> 5	50	28	22	
Tumor number				
single	98	45	53	0.313
multiple	59	32	27	
Tumor encapsulation				
complete	72	35	37	0.920
none	85	42	43	
Vascular invasion				
no	112	61	51	0.032
yes	45	16	29	
BCLC stage				
0-A	99	56	43	0.014
B-C	58	21	37	
Lung metastasis				
no	134	67	67	0.566
yes	23	10	13	
Lymph node metastasis				
no	137	68	69	0.701
yes	20	9	11	
Bone metastasis				
no	139	74	65	0.003
yes	18	3	15	

*HBsAg* hepatitis B surface antigen, *HCV-Ab* hepatitis C virus antibody, *AFP* a-fetoprotein, *ALT* alanine aminotransferase, *γ-GT* γ-glutamyl transferase, *BCLC-stage* Barcelona Clinic Liver Cancer-stage

At the final follow-up, 50 (31.8%) patients were found to have extrahepatic metastases. Among these patients, 23 patients developed lung metastasis, 20 patients developed lymph node metastasis, and 18 patients developed bone metastases. To eliminate the possibility of other extrahepatic metastases affecting the expression of lnc34a, we further carried out a subgroup analysis of its expression with other extrahepatic metastases. Correlation analysis revealed that the serum lnc34a expression level was positive correlated with bone metastasis in HCC (*r* = 0.233, *p* = 0.003), while there was no significant correlations between circulating lnc34a expression and lung (*r* = 0.046, *p* = 0.566) or lymph node metastasis (*r* = 0.031, *p* = 0.701).

### Cox regression analysis of potential biomarkers and bone metastasis

As shown in Table 3, univariate analyses indicated that vascular invasion (*p* < 0.001), and BCLC stage (*p* = 0.001) and lnc34a expression (*p* = 0.008) were significantly associated with HCC bone metastasis. However, there was no significant correlation between bone metastasis and clinicopathological factors such as age, gender, HBsAg, HCV-Ab, AFP, ALT, γ-GT, liver cirrhosis, Child-Pugh score, tumor differentiation, tumor size, tumor number and tumor encapsulation. Multivariate analyses further revealed that vascular invasion (95% confidence interval [CI], 2.291–19.157; *p* < 0.001), BCLC stage (95% CI, 1.244–12.287; *p* = 0.020), and lnc34a expression (95% CI, 1.107–13.629; *p* = 0.034) were associated with bone metastasis (Table 4). Therefore, high serum lnc34a expression, vascular invasion and BCLC stage were independent risk factors for bone metastasis prediction in HCC patients.

**Table 3:** Univariate analyses of factors associated with bone metastasis in 157 HCC patients

Variable	Bone metastasis	
	HR (95% CI)	*P*
Age (≤56 versus > 56 years)	0.725(0.286–1.840)	0.499
Gender (male versus female)	1.864(0.539–6.445)	0.325
HBsAg (negative versus positive)	1.849(0.425–8.047)	0.412
HCV-Ab (negative versus positive)	1.707(0.227–12.838)	0.603
AFP, ng/mL (≤ 20 versus > 20)	1.612(0.574–4.526)	0.364
ALT,U/L (≤ 40 versus > 40)	0.255(0.034–1.915)	0.184
γ-GT,U/L (≤ 50 versus > 50)	1.032(0.400–2.662)	0.949
Liver cirrhosis (no versus yes)	4.662(0.620–35.039)	0.135
Child-Pugh score (A versus B)	1.155(0.153–8.697)	0.889
Tumor differentiation (I–II versus III–IV)	1.504(0.597–3.789)	0.387
Tumor size, cm (≤ 5 versus > 5)	0.657(0.216–1.996)	0.458
Tumor number (single versus multiple)	0.963(0.373–2.486)	0.937
Tumor encapsulation (complete versus none)	1.079(0.426–2.737)	0.872
Vascular invasion (no versus yes)	10.054(3.572–28.302)	< 0.001
BCLC stage (0-A versus B-C)	7.193(2.361–21.919)	0.001
Lnc34a (low versus high)	5.385(1.558–18.612)	0.008

*HCC* hepatocellular carcinoma, *HBsAg* hepatitis B surface antigen, *HCV-Ab* hepatitis C virus antibody, *AFP* a-fetoprotein, *ALT* alanine aminotransferase, *γ-GT* γ-glutamyl transferase, *BCLC-stage* Barcelona Clinic Liver Cancer-stage

**Table 4:** Multivariate analyses of factors associated with bone metastasis in 157 HCC patients

Variable	Bone metastasis	
	HR (95% CI)	*P*
Vascular invasion (no versus yes)	6.625(2.291–19.157)	< 0.001
BCLC stage (0-A versus B-C)	3.910(1.244–12.287)	0.020
Lnc34a (low versus high)	3.883(1.107–13.629)	0.034

*HCC* hepatocellular carcinoma, *BCLC-stage* Barcelona Clinic Liver Cancer-stage

## Discussion

Bone metastasis occurred in 25.5 to 38.5% of HCC patients with extrahepatic metastases [26] and in 11.7% of HCC patients after hepatectomy [23].The recurrent skeletal-related events (SREs) such as pain and fractures, severely affected the prognosis and quality of life of HCC patients [3]. Early prediction or detection of bone metastases is urgently needed in clinical practice to identify the best treatment for patients at high risk of bone metastasis and avoid the subsequent complications.

The search for specific disease-related biomarkers in patient samples including tissues and body fluids through molecular biology techniques is known as molecular diagnostics [27]. The use of biomarkers could contribute to early diagnosis of cancer, monitoring of disease, as well as the response of therapy, substantially improving patients’ survival rates and quality of life [27]. Our previous study has found that intratumoral connective tissue growth factor (CTGF) expression, interleukin-11 (IL-11) expression and the CXC chemokine receptor 4 (CXCR4) chemokine receptor may serve as useful predictive biomarkers for bone metastasis in HCC patients [23, 28]. We previously established a predictive model incorporating the tumor properties of vascular invasion and TNM stage, and CXCR4, CTGF, and IL-11 proteins to predict bone metastasis in HCC [29].

However, tissue biopsies are clinically unfeasible for some patients [9]. Tissue biopsies are invasive examinations which may cause substantial complications in patients through biopsies or needle aspirations [30]. While liquid biopsy is a non-invasive procedure and allows for repeat sampling in serum, plasma or saliva [31, 32], which would be useful for numerous diagnostic and monitoring applications in cancer patients with advanced-stage, as is the case for bone metastases [33]. It can detect circulating tumor cells (CTCs), circulating free DNA (cfDNA), messenger RNA (mRNA), or microRNA (miRNA) which are released into the bloodstream by cancer cells [31]. Up to date, genome sequencing studies have identified that increased level of circulating lncRNAs has been observed in the blood of patients with cancer. LncRNAs becomes emerging in the search of novel biomarkers for diagnostics or prognostics in cancer due to its greater tissue specificity compared with protein-coding mRNAs [34]. For instance, the upregulated LINC00161 in serum samples of HCC patients was useful for early diagnosis of HCC [35]. A three-lncRNA panel (PCAT-1, UBC1 and SNHG16) in serum exosomes may serve as valuable diagnostic and prognostic biomarkers of bladder cancer [36]. What’s more, Duan, W et al. (2016) have confirmed that serum lncRNAs are very stable even incubated for a long time at room temperature or at − 80 °C and repeated freeze-thaw cycles [37]. The highly stability of circulating cell-free lncRNA may due to the protection by extracellular vesicles including exosomes, microvesicles and apoptotic bodies and the combination with protein or other chromatin remodeling complexes [37, 38].

Nowadays, lncRNAs have played an emerging role in bone metastasis of other cancers. Liu, M et al. demonstrated MALAT1 was significantly highly expressed in non-small cell lung cancer (NSCLC) tissues with bone metastasis and in NSCLC cell lines with high bone metastatic ability [39]. Li C et al. (2017) suggest that ROR1-HER3-LncRNA(MAYA) signaling axis modulates the Hippo-YAP pathway to regulate bone metastasis and serves as a promising therapeutic target for bone metastasis [40]. Liu P et al. (2018) indicated that LINC00852 promotes lung adenocarcinoma spinal metastasis by targeting S100A9 [41]. Although lncRNAs have played an emerging role in bone metastasis from other cancers, there are no studies assessing serum lncRNAs in cancer patients with bone metastasis.

Lnc34a was first discovered by Wang et al. [19] (2016). They originally wanted to identify what decreases miR-34a expression in the bowel cancer cells. Then they discovered the potential transcripts within miR-34a promoter region by RT-PCR with 10 pairs of primers and continued to amplify a 293 bp transcript fragment. Finally, a new long non-coding RNA molecule, named lnc34a with a full-length of 693 bp transcript, was identified by rapid amplification of cDNA ends (RACE) and further confirmed by norther blot assay in colorectal cancer cell lines and colon cancer stem cell lines. The uneven distribution of lnc34a in colon cancer cells division suppressed miR-34a expression in one of the daughter cells and then sped up cells division [19]. In the current study, we showed that the expression levels of serum lnc34a in patients with bone metastases were significantly higher than those without bone metastases. Analysis revealed that patients with high serum lnc34a expression were more likely to exhibit high rates of vascular invasion and advanced BCLC stage. In addition, correlation analysis revealed that lnc34a expression was significantly correlated with HCC bone metastases. Univariate and multivariate analysis identified that serum expression of lnc34a, vascular invasion and BCLC stage were significantly associated with bone metastasis. Besides, we determined that lnc34a expression levels were specifically associated bone metastasis rather than lung or lymph node metastasis. Our findings suggest that circulating lnc34a, vascular invasion and BCLC stage were independent risk factors for bone metastases in HCC patients. With the lnc34a expression, vascular invasion and BCLC stage, HCC patients were divided into low- or high-risk groups for bone metastasis. Therefore, HCC patients at high risk of bone metastases can be identified by analyzing circulating lnc34a expression and other clinicopathological factors. The prophylactic treatment such as oral clodronate, could be taken for the HCC patients who are at a high risk for bone metastasis to reduce the frequency and severity of skeletal complications. However, there are certain limitations in our study. This was a retrospective study and the population of enrolled patients was relatively small. In the future, more large-scale studies are needed to further confirm our findings. A detailed mechanism of how lnc34a affects HCC tumors cells and spread to bone should be further investigated.

## Conclusions

Based on the results of our study, the expression levels of serum lnc34a in HCC patients with bone metastases were significantly higher than those without bone metastases. Therefore, circulating lnc34a, vascular invasion and BCLC stage were independent risk factors for bone metastases in HCC patients.

## Supplementary Information

**Additional file 1: Supplementary figure 1.** ROC curves analysis of lnc34a expession.

## Data Availability

All data generated or analysed during this study are included in this published article. Additional data/files would be available from the corresponding author upon reasonable request.

## Abbreviations

HCC: Hepatocellular carcinoma
lncRNAs: Long noncoding RNAs
ESCC: Oesophageal squamous cell carcinoma
AFP: Alpha-fetoprotein
DCP: Des-gamma-carboxy prothrombin
CCSC: Colon cancer stem cells
qRT-PCR: Quantitative real-time PCR
HBsAg: Hepatitis B surface antigen
HCV-Ab: Hepatitis C virus antibody
ALT: Alanine aminotransferase
γ-GT: Gamma-glutamyl transferase
BCLC: Barcelona Clinic Liver Cancer
SREs: Skeletal-related events
CTCs: Circulating tumor cells
cfDNA: Circulating free DNA
mRNA: Messenger RNA
miRNA: microRNA
NSCLC: Non-small cell lung cancer
