# Supplementary figures and images for: Serum lnc34a is a potential prediction biomarker for bone metastasis in hepatocellular carcinoma patients
Source: BMC Cancer. 2021 Feb 15;21:161. doi: 10.1186/s12885-021-07808-6 (PMC7885499; doi:10.1186/s12885-021-07808-6)

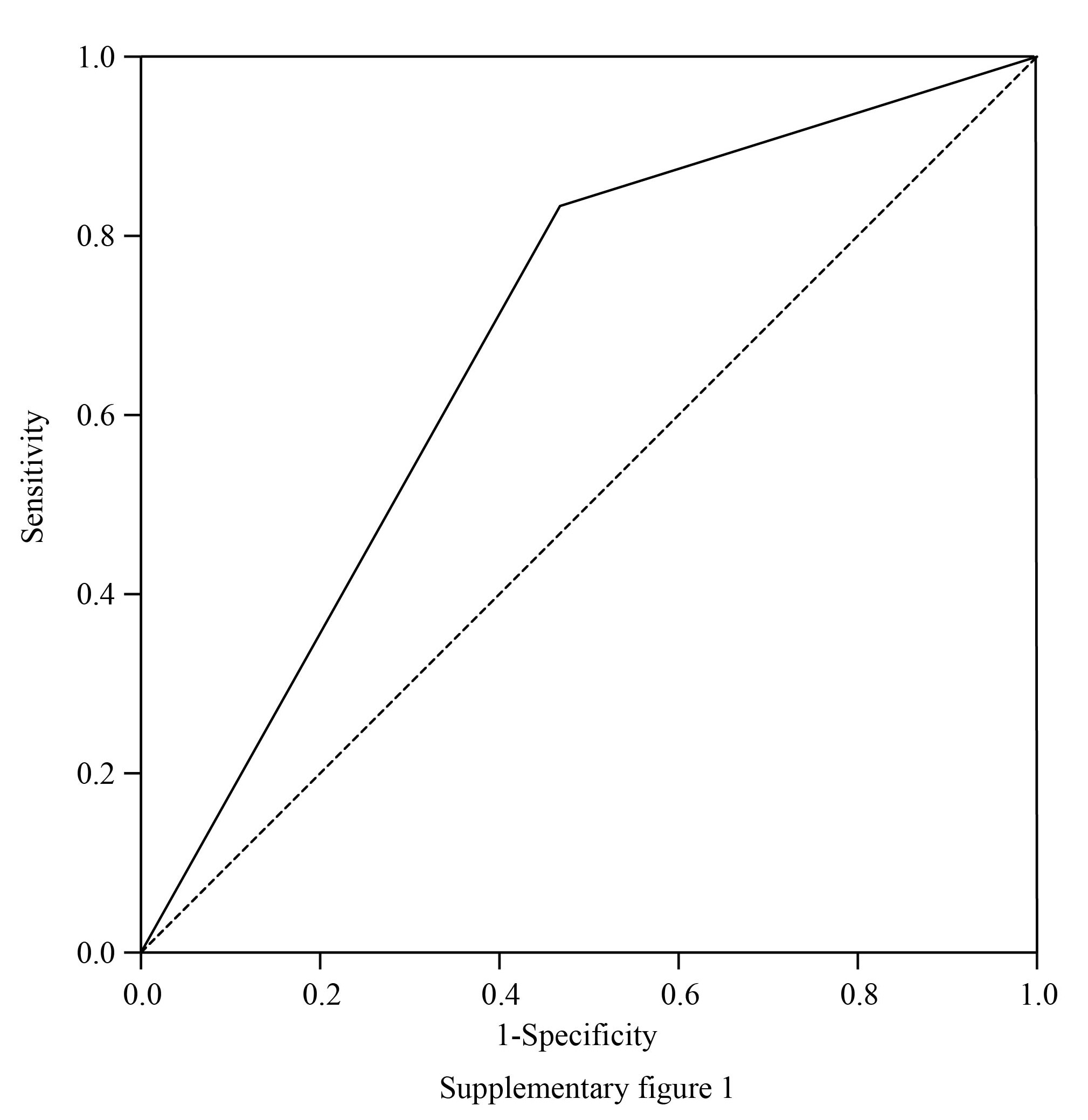

Supplement: Supplementary file 1 — Additional file 1: Supplementary figure 1. ROC curves analysis of lnc34a expession. [file 12885_2021_7808_MOESM1_ESM.jpg]
